# Utilisation of Deep Neural Networks for Estimation of Cajal Cells in the Anal Canal Wall of Patients with Advanced Haemorrhoidal Disease Treated by LigaSure Surgery

**DOI:** 10.3390/cells14070550

**Published:** 2025-04-05

**Authors:** Inese Fišere, Edgars Edelmers, Šimons Svirskis, Valērija Groma

**Affiliations:** 1 Department of Doctoral Studies, Rīga Stradiņš University, Dzirciema Street 16, LV-1007 Riga, Latvia; inese_fisere@inbox.lv; 2 Surgery Clinic, Pauls Stradins Clinical University Hospital, Pilsonu Street 13, LV-1002 Riga, Latvia; 3 Medical Education Technology Centre, Rīga Stradiņš University, Dzirciema Street 16, LV-1007 Riga, Latvia; 4 Faculty of Computer Science Information Technology and Energy, Riga Technical University, LV-1048 Riga, Latvia; 5 Institute of Electronics and Computer Science, Dzerbenes Street 14, LV-1006 Riga, Latvia; 6 Institute of Microbiology and Virology, Rīga Stradiņš University, Ratsupītes Street 5, LV-1067 Riga, Latvia; simons.svirskis@rsu.lv; 7 Institute of Anatomy and Anthropology, Rīga Stradiņš University, Dzirciema Street 16, LV-1007 Riga, Latvia

**Keywords:** interstitial cells of Cajal, deep neural networks, advanced haemorrhoidal disease, LigaSure surgery, anoctamin 1

## Abstract

Interstitial cells of Cajal (ICCs) play a key role in gastrointestinal smooth muscle contractions, but their relationship with anal canal function in advanced haemorrhoidal disease (HD) remains poorly understood. This study uses deep neural network (DNN) models to estimate ICC presence and quantity in anal canal tissues affected by HD. Haemorrhoidectomy specimens were collected from patients undergoing surgery with the LigaSure device. A YOLOv11-based machine learning model, trained on 376 immunohistochemical images, automated ICC detection using the CD117 marker, achieving a mean average precision (mAP50) of 92%, with a recall of 86% and precision of 88%. The DNN model accurately identified ICCs in whole-slide images, revealing that one-third of grade III HD patients and 60% of grade IV HD patients had a high ICC density. Preoperatively, pain was reported in 35% of grade III HD patients and 41% of grade IV patients, with a significant reduction following surgery. A significant decrease in bleeding (*p* < 0.0001) was also noted postoperatively. Notably, patients with postoperative bleeding, diagnosed with stage IV HD, had high ICC density in their anorectal tissues (*p* = 0.0041), suggesting a potential link between ICC density and HD severity. This AI-driven model, alongside clinical data, may enhance outcome prediction and provide insights into HD pathophysiology.

## 1. Introduction

The current understanding of haemorrhoidal development suggests that it results from a multifaceted interplay of anatomical and physiological alterations, including the displacement of the anal cushions due to disruption of the fixation network, complex changes within the vascular structures of the anorectal region such as venous distension and altered haemodynamic profiles, distension and subsequent vascular congestion caused by excess tissue, and increased intra-abdominal pressure, which places heightened strain on the anorectal vascular plexus [1].

The prevalence of haemorrhoids ranges from 38.9% to 44.7% in colonoscopy screening, whereas the incidence is around 55% [2,3,4,5]. The recurrence rate with nonsurgical techniques is 10–50% over a five-year period, whereas with haemorrhoidectomy, it is less than 5% [3]. A haemorrhoidectomy is the gold-standard treatment for patients with grade III or grade IV haemorrhoidal disease (HD), offering the lowest recurrence rate [3,4,6,7,8,9,10]. The most serious complication is faecal incontinence due to damage to the sphincter, although haemorrhoidectomy can cause changes in continence without direct sphincter injury [6,11]. After excisional haemorrhoidectomy, patients typically have linear wounds that may lead to internal anal sphincter (IAS) hypertonia [10]. Postoperative pain and tenesmus are commonly attributed to increased muscle spasms caused by the surgical wound, often necessitating physiotherapist-led interventions or the addition of neurostimulation for neuromuscular therapy [12].

LigaSure haemorrhoidectomy (LH) is a closed procedure without sutures, using the instrumental sealing of mucosal edges and dividing the pedicle [3]. It reduces reflex anal spasm, allows for bloodless haemorrhoidectomy, accelerates healing, and reduces postoperative pain, often caused by injury or sutures to the IAS [3,6,10]. In LH, the advanced system identifies tissue type between the instrument’s jaws, applying the right pressure and energy to seal the vessel wall with minimal tissue damage, proving more effective in reducing postoperative pain and complications than traditional haemorrhoidectomy [3,7,13].

In the gastrointestinal (GI) tract, interstitial cells of Cajal (ICCs) act as natural pacemakers, generating bioelectrical slow waves that trigger electrical activity and regulate gut motility [14,15,16]. This pacemaker function, involving the generation of calcium waves, triggering rhythmic contractions, and establishing basal tone, has been previously proven for the IAS [17]. ICC pacemaker activity is thought to arise from intracellular Ca^2+^ release from the smooth endoplasmic reticulum, activating anoctamin 1 (ANO1), a calcium-activated chloride channel [17,18].

The substantial progress made in artificial intelligence (AI) over the past decade has the potential to significantly impact clinical practice. Furthermore, recent AI-guided applications have been developed to enhance the morphological analysis of various upper and lower GI tract pathologies, including inflammatory conditions and tumours [19,20,21,22,23]. Moving beyond these well-recognised and explored pathologies, we propose applying AI to a specific anatomical region of the GI tract—the anal canal. In our study, the use of an artificial neural network structured as a deep neural network (DNN) aimed to map the tissue distribution of ICCs, thereby improving the understanding of the underlying ICC pacemaker function and coupling these findings with the investigation of clinical data and LH surgical outcomes using extensive bioinformatics. This novel approach specifically targets the results of LH surgery, emphasising the need for an in-depth analysis of the pathobiology of HD, particularly when addressing the major complaints and treatment strategies.

The primary objective of this study was to determine the capacity of AI, specifically the integration of DNN into the pathology workflow, and its application in the IHC analysis of anal canal tissues removed during LH intervention. Additionally, it aimed to link these data to the assessment of surgical outcomes in patients with advanced stages of HD.

## 2. Materials and Methods

### 2.1. Study Design

In this retrospective cohort study, forty-two patients were included who underwent excisional haemorrhoidectomies with the LH device at Pauls Stradiņš Clinical University Hospital, Riga, Latvia, from January 2021 to December 2022. The database used for patient selection was comprehensive and included HD patients from a regional medical centre, thus enhancing the representativeness of the study population. This study was approved by the Ethics Committee of Riga Stradiņš University (Decision No. 22-2/264/2021) and conducted in accordance with the Declaration of Helsinki. All sensitive information was excluded, and informed consent was obtained from all study participants.

The inclusion criteria for the study cohort were patients over 18 years of age with haemorrhoidal disease grade III or IV, with indications for excisional haemorrhoidectomy, identified with an American Society of Anaesthesiologists (ASA) score of I or II. The exclusion criteria were patients with concomitant pathologies of the anal region (sphincter defect, rectal prolapse, polyps, inflammatory diseases, etc.), previous operations for anal incontinence, existing incontinence for solid stool, identified with an ASA score of III, or a planned duration of hospitalisation due to general illnesses exceeding three days. The rationale for excluding these pathologies was to obtain more objective data on the healing process in conjunction with LigaSure surgery for HD and to ensure a proper understanding of the postoperative clinical manifestations that occur thereafter.

The following clinical parameters were recorded, collected, and analysed: age, sex, presenting symptoms, complaints, surgery description, immediate postoperative complications, and visit conclusions. Pain intensity was assessed using the Visual Analogue Scale (VAS). Postoperative digital and anoscope examinations were carried out during follow-up visits for six weeks, with a final control visit 9 months after surgery.

### 2.2. Surgical Procedure

The procedure was performed using an LH device. Excisional haemorrhoidectomies were carried out for symptomatic prolapsed haemorrhoids, with an optimised combination of pressure and radiofrequency at positions 5, 7, and 11 o’clock. The patient received premedication, analgesia, and 500 mg of metronidazole preoperatively. Under regional anaesthesia, in the lithotomy position, a 5 mm “V” incision was made with scalpel No. 11 at the border of the anal canal and the skin using an anoscope. The nodule was retracted, and the LH was placed 3 mm above the sphincter at the base of the haemorrhoidal nodule and resected without any sutures. Haemostasis control was then carried out, if necessary, using electrocoagulation. A swab with lidocaine-containing gel was inserted into the rectum for up to 24 h after surgery. Patients were discharged the next day after wound control, with analgesic and flavonoids, and analgesia and granulation-enhancing ointments.

### 2.3. Immunohistochemistry Procedure

Forty-two formalin-fixed and paraffin-embedded (FFPE) HD anorectal tissue samples were collected retrospectively, sectioned, and mounted on SuperFrost Plus slides (Gerhard Menzel GmbH, Braunschweig, Germany). Haematoxylin and eosin staining confirmed the diagnosis of HD and identified the tissue site relative to the dentate line and mucosal epithelial type. CD117, used as the primary protein marker of ICCs in pathology specimens, and ANO1, also known as TMEM16A, expressions were assessed immunohistochemically according to the manufacturer’s guidelines. Deparaffinised sections were incubated overnight at 4 °C with a rabbit monoclonal antibody against CD117 (c-kit) (YR145) (Cell Marque, Rocklin, CA, USA, 1:300 dilution) and a rabbit polyclonal antibody against TMEM16A (Abcam, Cambridge, UK, 1:100 dilution, ab201980). The IHC reactions were visualised using a HiDef Detection HRP Polymer system and a diaminobenzidine substrate kit (Cell Marque, Rocklin, CA, USA), with Mayer’s haematoxylin counterstaining cell nuclei. Negative controls omitted the primary antibodies. The immunostaining was conducted on all tissue slides at once to achieve objective results from IHC performance, avoiding multiple repetitions. The evaluation of the results was performed by two experienced morphologists who were blinded to the clinical and histopathological data. Images were captured with a Glissando Slide Scanner (Objective Imaging Ltd., Cambridge, UK). ANO1 expression was assessed using semiquantitative scoring: 0 for negative expression, 1 for 1–10%, 2 for 11–50%, and 3 for over 50%, in both epithelial cells of the crypt and myocytes.

### 2.4. Training and Validation Cohorts

Models of varying architecture sizes were trained and optimised to enhance performance and efficiency. Through careful simplification, each model was refined to balance accuracy with computational demands. A total of 40 patches, each with a resolution of 2048 × 2048, were extracted from whole slide images (WSIs), depicting immunohistochemically detected ICCs. These images were initially obtained at maximum magnification. To align with the recommended patch size of the YOLOv11n-obb architecture, each image was subsequently divided into smaller 1024 × 1024 sections, producing a total of 160 images. All images were annotated, resulting in 1871 labelled masks for training purposes.

YOLOv11 is among the newest architectures offering oriented object detection capabilities. Oriented object detection extends conventional methods by incorporating an additional angular parameter, which enables the more precise localization of objects within an image. Moreover, it has been demonstrated to achieve state-of-the-art performance in terms of mAP50 and processing speed on the standard COCO dataset [24].

The rotation and flipping augmentation techniques were employed to enhance the trained model’s robustness and mitigate potential performance variations due to differing immunostaining intensities. The final dataset comprised 376 images for training, with an additional set of 32 non-augmented images used for validation. Images representing all possible variations in the anorectal tissue, including mucosal epithelium, connective tissue, as well as both compact and loose muscular components, tissue regions distorted by dilated and disrupted vessels, and images depicting diverse distributions of ICCs, were selected. This selection process was consistently applied to both the training and validation sets.

The validation was performed by evaluating the model on a held-out validation set at the end of each training epoch. During this phase, the model performs inference on all validation images, and its predictions are compared to the corresponding ground truth annotations. Non-maximum suppression is applied to eliminate redundant bounding boxes, ensuring that each object is detected only once. The evaluation process calculates key metrics such as precision, recall, and mean average precision (mAP), specifically mAP50—where an Intersection over Union (IoU) threshold of 0.5 is used—and mAP averaged over a range of thresholds (mAP50-95). These metrics are aggregated across the entire validation set to provide a comprehensive assessment of the model’s performance on unseen data, guiding adjustments in hyperparameters and serving as a checkpoint for selecting the best-performing model.

### 2.5. Statistics

Data analysis and visualisation were executed utilising GraphPad Prism 9.0 for MacOS (GraphPad Software, San Diego, CA, USA), Jamovi (version 2.4.12, The Jamovi Project, Sydney, Australia), and JMP 17 (SAS, Cary, NC, USA). Descriptive statistics for clinical parameters were represented as median values accompanied by their corresponding interquartile ranges (IQRs). For the comparative analysis of immunostaining values within HD patient groups, the non-parametric Wilcoxon signed-rank test was employed. The distribution of variables in the anorectal tissue of male and female patients with HD was assessed using Chi-squared analysis. To explore potential associations between patient symptomatology and demographic correlations with sex, Spearman’s rank correlation coefficient was calculated. Multivariate pattern recognition was performed through hierarchical cluster analysis using Ward’s minimum variance method, enabling the identification of distinct subgroups, the exploration of data similarities and differences, and the detection of latent data structures. The threshold for statistical significance was set at *p* < 0.05 for all analytical procedures.

## 3. Results

The median age was 53 years for women and 46 years for men, with a range of 24 to 72 years. Three women (15%) and seven men (31.81%) were under 40. Of the patients, 50% of women and 45.45% of men had HD stage III, while 50% of women and 54.55% of men had HD stage IV. Bleeding was the main preoperative complaint in both genders for HD stage III (75%), followed by prolapsed tissue (45%) with no significant gender differences. Men reported more faecal spotting (20%) and formations (20%) than women (10%). For HD stage IV, bleeding (72.7%) and formations (40.9%) were most common, with pain more frequent in women (38.4%) than men (4.5%). Males complained more of faecal spotting (18.2%) and discomfort (18.2%), while women reported itching and burning (18.2%). There were no significant gender differences in preoperative complaints across age groups. The average HD symptom duration was 4.6 years for women and 7.8 years for men. While women had more nodes excised, this was not associated with patient complaints.

**Figure 1:**
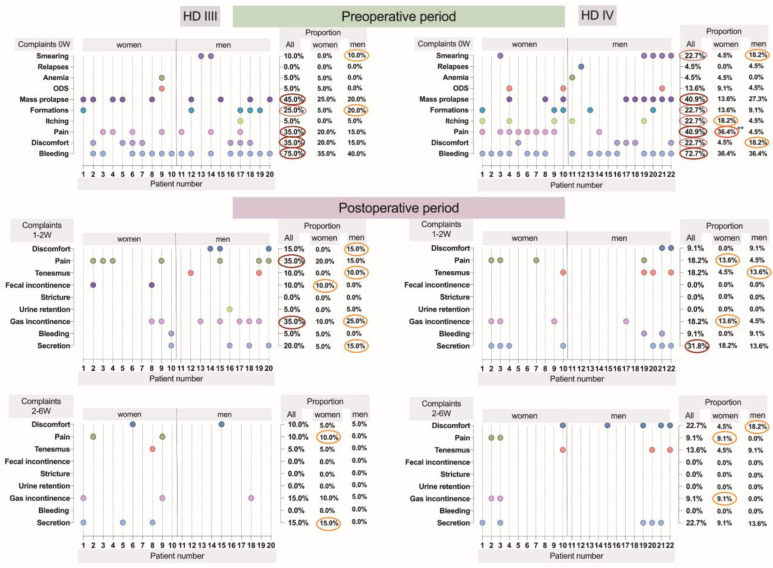
Dynamics of preoperative and postoperative complaints of study participants, along with disease characteristics.

At the first postoperative visit, 1–2 weeks after LH, discomfort and maceration were more pronounced in males (15%), with 25% reporting bleeding without defecation after haemorrhoidectomy for grade III HD compared to none after grade IV HD. Faecal incontinence occurred in 10% of males after LH for HD III but not in those with HD IV. Both grades provoked tenesmus in males, more so after grade IV (13.6%). In contrast, females showed fewer postoperative complaints, with 10% experiencing faecal incontinence after LH for HD III. After grade IV surgery, 13.6% of women had bleeding without defecation. Maximum pain (VAS score of 8) was reported by six (30%) women aged 43–66 years (mean age 56.8), one of whom had reduced anal sphincter tone preoperatively. In males, only two (9.09%) patients reported maximum pain, aged 30 and 52, with abnormal anal sphincter tone. Three males aged 27, 29, and 44 had persistent complaints beyond 6 weeks, including discomfort, oedema, and previous issues such as bleeding, tissue prolapse, faecal incontinence, discomfort/itching, peri-anal formations, and obstructive defecation. The research data regarding the complaints of study participants and the stage of HD have been summarised and are presented in Figure 1.

In the postoperative period, analgesia was administered to all patients, with Ultracod 500 mg/30 mg tablets (up to 4× daily) or Ketanov 10 mg tablets (up to 2× daily) being the most common. Flavonoids were prescribed in 76.2% of cases, while antibacterial therapy was indicated in 19%. Topical granulation-stimulating creams were used in 42.9% of patients, and diltiazem was applied topically in 28.6% of patients, with men receiving it twice as often. Physiotherapist supervision for pelvic muscle coordination was required in 16.7% of cases, with no significant gender difference. Neurostimulation for correct neuromuscular therapy was needed in 14.3% of patients, equally distributed across both sexes (Supplementary Figure S1).

In the “All” columns, dark red ovals represent the most pronounced complaints (>30%) in terms of percentage, while brown ovals indicate less pronounced complaints (20–30%). Orange ovals highlight complaints that are dominant in either women or men, whereas red ovals denote significant differences between the sexes, as determined by Fisher’s exact test (*p* < 0.05).

**Figure 2:**
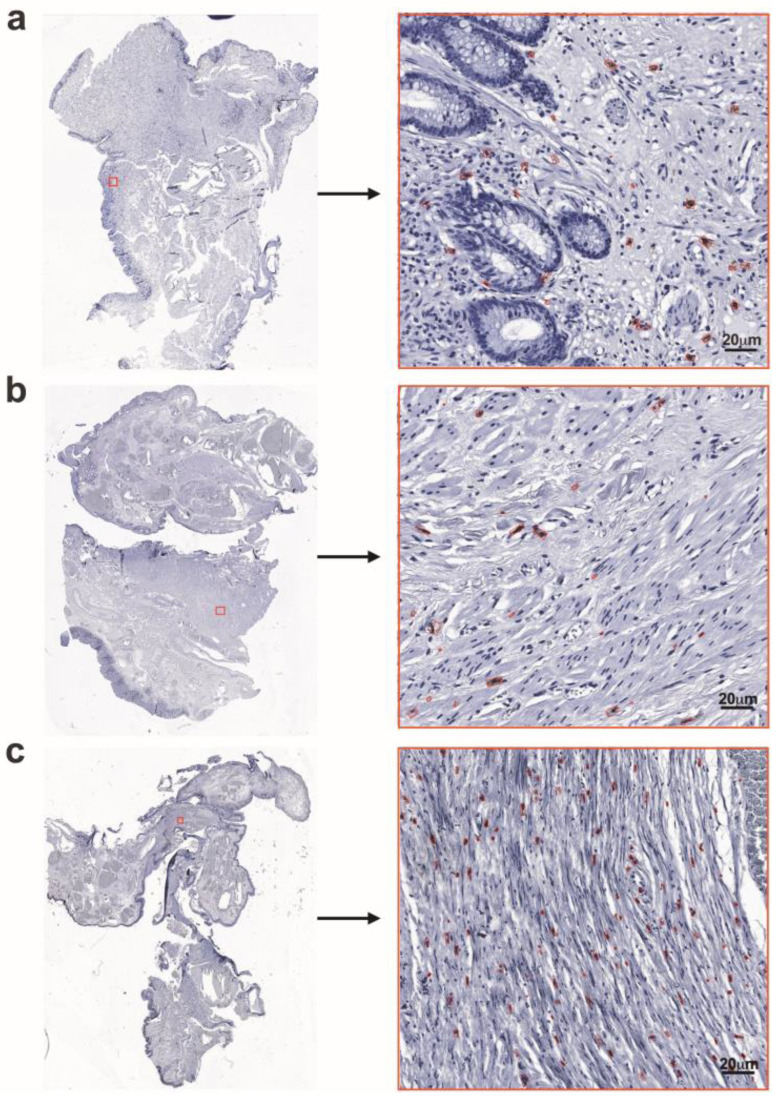
ICC IHC performed on FFPE HD anorectal tissue samples, processed from whole slide images with region selection and detection using DNN. (**a**) A representative image demonstrating the loosening of the mucosal muscular component with haphazard orientation of myocytes, with some ICCs displaying brown coloration. (**b**) A small number of ICCs in the muscularis externa, corresponding to DNN detection. (**c**) Densely distributed ICCs in the muscularis externa, as detected by DNN. Scale bars: 20 µm.

CD117 and ANO1 immunohistochemistry were employed to detect the presence and distribution of ICCs and to assess the membranous expression of ANO1, a calcium-activated chloride channel found in anal glandular and smooth muscle cells, respectively. Immunostaining revealed ICCs as ramified cells, dispersed among the smooth muscle cells. The density of ICCs associated with the muscular component of the anal canal wall, as estimated in the study cohort of patients with HD, varied significantly (Figure 2a–c). With the loosening of the muscular component of the anal canal wall and the appearance of large, dilated vessels, the ICCs demonstrated a reduction in number and were often localised perivascularly.

ANO1 immunoreactivity was predominantly observed at the membrane of anal gland epithelial and smooth muscle cells (Supplementary Figure S2). The expression of ANO1 across the specimens in the study cohort varied considerably, with some membranes showing no expression, while others were assessed semiquantitatively with a score of “3”, indicating appropriate expression in more than 50% of the structures.

**Figure 3:**
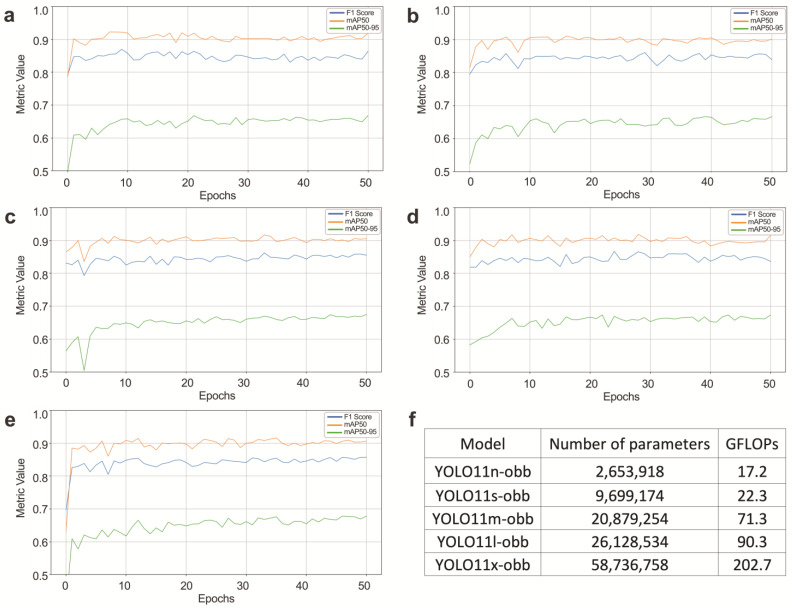
Performance of the trained models. (**a**) YOLO11n-obb, (**b**) YOLO11s-obb, (**c**) YOLO11m-obb, (**d**) YOLO11l-obb, (**e**) YOLO11x-obb, and (**f**) parameters of the trained models.

Several models of varying sizes were trained to detect ICCs, as shown in Figure 3. Training was conducted over 51 epochs, evaluating model performance using three primary metrics: mAP50, mAP50-95, and F1 score. All DNN models exhibited similar performance, achieving a mean average precision at 50% (mAP50) of 92%, with a recall of 86% and a precision of 88%, which is deemed adequate for cell-counting tasks. The model selected for integration prioritised efficiency, balancing low parameter count for improved performance on low-powered devices and faster inference speed. The final model chosen for implementation was YOLOv11n-obb, optimised for both accuracy and resource efficiency [24]. The surface area quantification module within the programme provided a method for assessing histological sections and delineating specific tissue regions. Utilising either the resolution metadata embedded within TIFF files or a user-defined pixel size, the algorithm performs pixel-to-area conversions in square millimetres. Through the application of morphological operations, the programme generates a tissue mask, facilitating the computation of the tissue-occupied area relative to the entire histological slide. Following model training, the “MorpHista” (1.0) software was developed to efficiently process large WSIs and quantify ICCs. It integrated automated detection and post-processing, addressing the challenge of reassembling segmented regions after inference to consolidate all detected features into a single image file while compiling relevant statistics, including ICC counts. The tool has been made publicly available, enabling reproducible ICC analysis and fostering broader research innovation in digital pathology.

Despite the efforts to incorporate a segmentation approach for quantifying ANO1 expression, we did not achieve our targeted Dice coefficient. This shortfall highlights the complexity inherent in pixel-level classification tasks, especially given the variability in staining intensities and tissue morphology within our dataset. A robust linear regression analysis simultaneously confirmed an inverse relationship between the number of ICCs and ANO1 expression in anorectal tissue of patients with advanced HD. Tissue samples with reduced ANO1 expression in myocyte membranes showed a higher number of ICCs, whereas samples with elevated ANO1 expression exhibited a lower number of ICCs (Supplementary Figure S3). However, further hierarchical clustering analysis revealed other important trends (Supplementary Figure S5). It confirmed that HD patients in the green cluster, as well as most in the blue cluster, exhibited low to moderate ICC density in their samples, which corresponded with low ANO1 expression. In contrast, patients in the orange cluster predominantly displayed high ICC density alongside elevated ANO1 expression. Finally, patients in the red cluster demonstrated highly heterogeneous patterns for both ICC density and ANO1 expression, including both similar and inverse correlations. Collectively, these findings, coupled with the inherent complexity of HD biology and the influence of individual patient characteristics identified in this analysis, provide an important context for understanding the results.

To gain a deeper understanding of the contribution of ICCs to the development of HD, we performed a correlation analysis of the estimations obtained using the AI-driven approach with surgically removed anal canal tissues, exploring the association between the number of counted ICCs and the tissue area of the anal canal wall. The estimated density of ICCs across surgical samples of various shapes and sizes is presented in Supplementary Figure S4. A 95% confidence interval (CI) for the mean calculated from the sample ranged from 0.3900 to 0.7849 (*p* < 0.0001).

**Figure 4:**
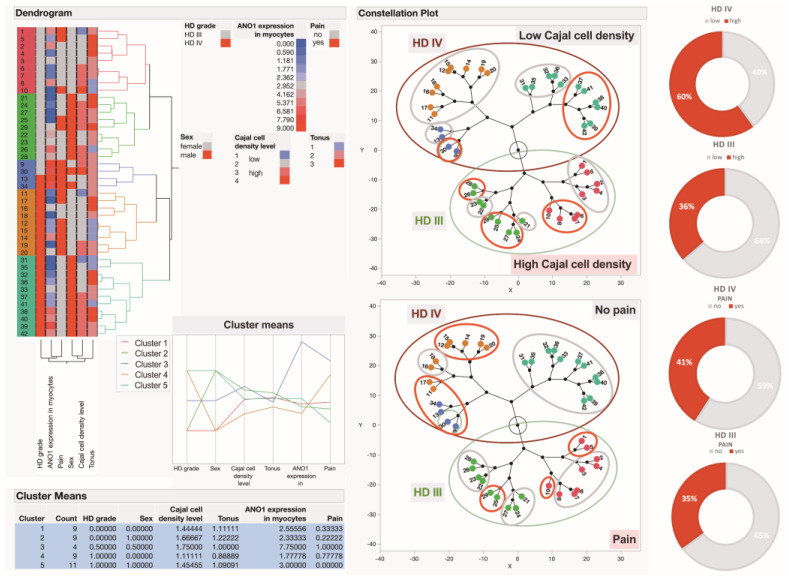
Hierarchical clustering of HD patients and visual representation of ICC density, pain manifestation, and disease severity in the study cohort. The dendrogram, as the output of the hierarchical clustering algorithm, represents a two-dimensional tree structure that depicts the order of nested clusters within the studied HD patient cohort, with respect to ICC density, pain manifestations, muscular tone, ANO1 expression levels in myocytes, patient sex, and disease severity. Variables are colour-scaled from blue to red, representing a gradient from the lowest to the highest observed values. The dendrogram demonstrates that distinct clusters of HD patients were identified primarily based on disease grade, patient sex, as well as ICC density and the presence of pain. Patients in the red, yellow-green, and one half of the blue-coloured clusters presented with HD grade III, whereas the remainder of the study cohort had grade IV. Notably, HD patients grouped in the blue-green-coloured cluster and most of the red-coloured cluster presented without pain, whereas HD patients in the blue-coloured cluster and half of the orange-coloured cluster manifested with pain. All HD males identified in the blue-green-coloured cluster, and almost all females in the red-coloured cluster, presented without pain.

Finally, in conducting an in-depth analysis of ICC estimation across the study samples, we aimed to enhance the understanding of the pathophysiology of HD. To this end, we applied an unsupervised clustering method to the research data, incorporating clinical data, IHC data, and DNN-derived findings related to the quantification of ICCs, which constituted either five (Supplementary Figure S5) or six (Figure 4) major factors included in the analysis. Of the patients, 43% of women and 57% of men had HD stage III, while 52% of women and 48% of men had HD stage IV. The ICCs appeared to be almost equally densely distributed across two large clusters of HD patients, characterised by disease grades III and IV, constituting 58% and 57%, respectively, of the anorectal tissues obtained from patients with HD grades III and IV (Supplementary Figure S5). Upon conducting the cluster analysis based on the exploration of six factors, only one-third (36%) of patients with HD grade III exhibited a high density of ICCs in their surgical specimens. In contrast, 60% of patients with HD grade IV showed a high density of these cells. For both stages of HD, discomfort, pain, and bleeding were observed both pre- and postoperatively. Specifically, preoperatively, pain was reported in 35% of patients with grade III HD and 41% of those with grade IV; however, it was markedly reduced following surgical treatment for stages III and IV HD. Regarding bleeding, a statistically significant reduction (*p* < 0.0001) in this symptom was observed postoperatively. However, two patients who experienced postoperative bleeding were diagnosed with stage IV HD and exhibited a high density of ICCs in their anorectal tissues (*p* = 0.0041), suggesting a potential association between ICC density and the severity of HD.

Using hierarchical clustering analysis, groups with similar characteristics were identified, with characteristics within a specific cluster being more like each other than those across the five clusters recognised in this study. The hierarchical clustering dendrogram shown in Figure 4 illustrates the formation of distinct patient clusters within the HD cohort. The constellation plots further illustrate the clustering of HD patients based on the varying levels of the respective variables. Additionally, the pie charts provide a visual representation of the disparities between patients with grade III and IV HD, categorised by low and high ICC density, alongside the differentiation between those manifesting with and without pain.

## 4. Discussion

HD is one of the most prevalent anorectal conditions encountered in general practice, affecting approximately one-third of the global population [6,25]. It is a leading contributor to lower GI bleeding and is recognised as a significant cause of morbidity [26]. It is a multifaceted condition that often carries significant emotional strain, necessitates daily adjustments, and has a considerable social impact. Common symptoms such as bleeding and pain frequently cause patients to experience fear and embarrassment, leading them to limit or avoid social activities. While some patients manage to adjust their daily routines to cope with the condition, many seek medical advice for treatment. Modern advanced surgical techniques for managing haemorrhoids, such as stapled haemorrhoidopexy, LigaSure excision, and haemorrhoidal artery ligation, generally yield positive outcomes. However, these procedures have become increasingly costly compared to traditional interventions, resulting in economic and social challenges within the community [27,28]. Therefore, research into surgical interventions that reduce tissue trauma, minimise incontinence, and improve wound healing without complications has been conducted.

Modern treatment methods for HD typically reduce both the duration of surgery and the recovery time required for patients to return to normal daily activities, compared to conventional surgical techniques. LH and Doppler-guided ligation of the haemorrhoidal arteries each have their own advantages and disadvantages. The reported length of hospital stays, postoperative bleeding time, and post-defecation pain score are significantly higher with LH. In contrast, the amount of bleeding during the operation is greater with vascular ligation [29]. As a bipolar electrosurgical instrument, the LigaSure device meets these requirements by achieving local tissue haemostasis through the denaturation of collagen and elastin within the vascular wall and surrounding connective tissue [30,31]. Haemorrhoidectomy performed with a vascular sealing device and the use of topical calcium channel blockers have demonstrated excellent results in reducing postoperative pain [32,33].

The complication rate following haemorrhoidectomy is generally proportional to the grade of the disease and the invasiveness of the surgery [34]. Postoperative pain presents a significant challenge after haemorrhoidectomy. In our study cohort, 35% of patients with grade III HD and 41% of patients with grade IV HD reported pain preoperatively, but this symptom was alleviated after surgery, suggesting the success of the applied treatment. The causes of post-haemorrhoidectomy pain are multifactorial, including the choice of surgical technique, the wound healing process, spasm of the anal sphincters, spasm of the *musculus puborectalis*, the administration of postoperative analgesia, stool consistency, and other factors [11,32,35]. While external sphincter spasms are typically mild and transient, IAS spasms can persist for longer. These spasms may result from exposure of the IAS myocytes during surgery, irritation by faeces, or involvement in suture bites. If the IAS spasm persists after the excised area heals, it may lead to an anal fissure, causing long-term pain following haemorrhoidectomy [36].

Several approaches can help reduce post-haemorrhoidectomy pain, such as the selection of the most suitable yet least traumatic surgical technique, the use of intraoperative adjuncts, anaesthetic methods, and postoperative interventions. Analgesics have been reported to provide the effective management of post-haemorrhoidectomy pain, regardless of whether they are administered locally or systemically [32,36]. These were applied to all study participants in our research (100% of cases). The second most used medication during the postoperative period was the application of phlebotonics, administered in two-thirds of all HD cases. These findings align with the results reported by other authors [37,38]. The topical application of diltiazem has been reported to effectively relieve pain after haemorrhoidectomy [32,33]. It acts by inhibiting the flow of extracellular calcium ions into the cisternae of the smooth endoplasmic reticulum in IAS myocytes, thereby preserving oxygen, which results in muscle relaxation and pain relief [33]. Previous studies have demonstrated that calcium channel blockers induce the relaxation of smooth muscle cells in the GI tract, and oral diltiazem has been shown to reduce resting anal pressure [32,33]. Almost one-third of all HD patients included in this study were treated with diltiazem.

Pain in HD implicates spasm of the IAS and the functioning of ICCs; however, it does not establish causality for this association. These are ubiquitous cells found between and within the smooth muscle layers of the digestive tract, from the oesophagus to the IAS in humans [39]. They exhibit CD117 (c-kit) immunopositivity and display morphological heterogeneity [40]. Previous studies have demonstrated that, in patients with obstructed defecation syndrome, ICCs are more densely distributed in the anterior wall of the rectum compared to the posterior wall and are more abundant than those in the controls, where ICCs are more evenly distributed, with their density being lower in the IAS [41,42]. In gastrointestinal inflammatory conditions, the number of ICCs is reduced, and ICCs exhibit abnormal ultrastructure and impaired function, which strongly suggests that gastrointestinal dysmotility in these diseases is partly due to ICC injury [43]. Furthermore, a link between ICCs and gastrointestinal stromal tumours (GISTs), which share similar markers indicating a common origin, has been suggested. ICCs, undergoing genetic alterations similar to those in GISTs, may act as precursors or provide a supportive microenvironment for GIST growth [44]. ICCs are electrically active cells that generate and transmit slow waves along the digestive tract, coordinating smooth muscle contractions and peristalsis [45,46]. However, under the influence of local tissue factors activating paracrine mechanisms, this pacemaker function can become excessive, leading to the creation of an ectopic pacemaker site that may initiate abnormal contractions [47,48,49,50]. ANO1, a Ca^2+^-activated Cl^−^ channel associated with the pacemaker function of ICCs, is an important molecule responsible for the mechanism of slow waves [47,48,49]. Unfortunately, under the conditions set in this investigation, we were unable to demonstrate significant associations between the expression of this plasma membrane protein and the ICC marker. Further research, incorporating individual features of HD patients and involving a larger cohort, may provide deeper insights into the complex biology of the disease [51,52]. Distributed within the smooth muscle layers, ICCs play a key role in regulating rhythmic muscle contractions [53]. This function is also facilitated by their mechanosensitive properties and the functioning of sodium channels, which enable the transduction of inhibitory and excitatory motor neuron inputs [54,55]. ICC dysfunction can cause motor abnormalities in conditions such as slow transit constipation, chronic idiopathic pseudo-obstruction, Hirschsprung’s disease, diverticular disease, and faecal incontinence [51,52]. The decline in ICC numbers, mainly due to the suppression of the signal-regulated kinase 1/2 signalling pathway, has also been observed alongside the ageing process and contributes to impaired fundic relaxation and decreased gastric compliance [56]. However, the specific role of ICCs in disease is not fully understood, highlighting the need for their detailed characterisation.

ICCs and their function have received significant attention from researchers across various medical subfields, with a range of methodologies, including computational approaches and mathematical modelling, employed for the precise quantification of these cells [57,58]. So far, most AI studies have focused predominantly on clinical and imaging modalities rather than on biological tissue data exploration [59]. AI-based methods have demonstrated considerable success in detecting various tumours and inflammatory conditions of the GI tract, contributing to the development of objective scoring systems for risk stratification, and being used to predict disease prognosis, including patient survival and treatment response [60,61]. However, to our knowledge, no research to date has employed a DNN-based model specifically to detect and quantify ICCs using tissue characteristics observed after immunohistochemical labelling. Notably, the recent systematic review and meta-analysis by McGenity et al. synthesised findings from 100 AI-driven pathology studies (48 included in the meta-analysis) and reported mean sensitivities and specificities of 96.3% (95% CI 94.1–97.7) and 93.3% (95% CI 90.5–95.4), respectively [31]. Our detection approach, characterised by an mAP50 of approximately 92%, falls within this high-accuracy range. These findings suggest that our model achieves performance comparable to recently developed AI systems in digital pathology, highlighting the versatility and diagnostic potential of DNN-based approaches for a wide range of histopathological applications.

In this research, we demonstrate that morphologically based DNN models can effectively integrate with clinical patient assessments to accurately predict surgical outcomes for HD patients. The models were trained and validated to identify the specific immunohistochemical marker CD117, which is characteristic of ICCs. This focus on ICCs is crucial due to its significant role in the pathophysiology of HD. We emphasised that a deeper understanding of the pathogenetic mechanisms of the disease, facilitated using AI, in conjunction with the appropriately selected and applied surgical technique, along with the use of appropriate pharmacological therapy and interventions during the postoperative period, can contribute to achieving better clinical outcomes.

The strengths of this study lie in our integration of DNN-based actions into the pathology workflow, particularly in relation to efficient WSI analysis. Additionally, we focused on cells that contribute to various GI tract diseases, enhancing the relevance and impact of our approach.

A primary limitation of our study is the moderate sample size of the cohort. Nevertheless, individuals with advanced haemorrhoidal disease (HD) were equally represented in grades III and IV, and the study included both sexes, as assigned at birth. Despite this constraint, the findings adequately reflect the characteristics of the investigated cohort, satisfying internal validity criteria through validation by a single evaluator. Another limitation is the narrow range of staining variability, as the current model was optimised for a specific local laboratory staining protocol. This constraint could be addressed by retraining the model with additional augmentation techniques—such as adjustments to hue, intensity, brightness, and contrast—to enhance its generalisability. Furthermore, compiling a training dataset that incorporates images from multiple sources, and then validating against both internal and external datasets, would further mitigate issues related to interobserver variability.

## 5. Conclusions

Surgical excision remains the most effective treatment for grade III and IV HD, offering the best long-term patient satisfaction despite an increased risk of initial side effects and complications. The AI-driven model employed in this study, focused on the histological features of HD with a particular emphasis on ICCs, when integrated with clinical data, proves to be a valuable tool for enhancing the understanding of disease pathophysiology. This approach also facilitates more accurate estimations of patient outcomes, thereby holding significant potential for both diagnostic and prognostic applications in clinical practice.

Furthermore, a dedicated application was introduced for WSI analysis and automated ICC quantification, seamlessly integrating tissue segmentation, cell- and tissue-level measurements, and the efficient handling of large TIFF files within a single platform. This software automatically generates annotated images, tissue masks, and summary reports, providing a thorough yet flexible tool for pathologists and researchers engaged in histopathological imaging.

## Data Availability

All images utilised for model training in this study are publicly available. The complete dataset has been deposited in the Zenodo repository and can be accessed via the following DOI: https://doi.org/10.5281/zenodo.14900511. Additionally, the software developed to implement and test the proposed model is openly available on GitHub at https://github.com/edlmar/MorpHista (accessed on 4 April 2025).

## Supplementary Materials

The following supporting information can be downloaded at: https://www.mdpi.com/article/10.3390/cells14070550/s1, Figure S1: Overview of postoperative medication regiments and interventions in patients with HD. Figure S2: ANO1 IHC performed on FFPE anorectal tissue samples from patients with HD. Figure S3: Association between the number of detected ICCs and ANO1 expression in anal canal wall myocytes. Figure S4: Association between the number of detected ICCs and the tissue area of anal canal wall specimens. Figure S5: Hierarchical clustering of HD patients and visual representation of ICC density, patient sex, and disease severity in the study cohort.

## Abbreviations

The following abbreviations are used in this manuscript:

AI	Artificial intelligence
ANO1	Anoctamin 1
ASA	American Society of Anaesthesiologists
CI	Confidence interval
DNN	Deep neural network
FFPE	Formalin-fixed and paraffin-embedded
F1	F1 Score (harmonic mean of precision and recall)
GI	Gastrointestinal
HD	Haemorrhoidal disease
HRP	Horseradish peroxidase
IHC	Immunohistochemistry
IQR	Interquartile range
LH	LigaSure haemorrhoidectomy
ML	Machine learning
mAP50	Mean average precision at 50% intersection over union (IoU)
mAP50-95	Mean average precision averaged over IoU thresholds from 50% to 95%
TMEM16A	Transmembrane protein 16A (alternative name for ANO1)
VAS	Visual Analogue Scale
WSI	Whole slide image(s)
TIFF	Tagged Image File Format

## References

[1] Lohsiriwat V., Ratto C., Parello A., Litta F. (2018). Anatomy, Physiology, and Pathophysiology of Hemorrhoids. Hemorrhoids. Coloproctology.

[2] Yuan C., Zhou C., Xue R., Jin X., Jin C., Zheng C. (2022). Outcomes of Modified Tissue Selection Therapy Stapler in the Treatment of Prolapsing Hemorrhoids. Front. Surg..

[3] Alhamdany A., Wahhab R.A.S.A., Lateef N.F. (2022). Ligasure^TM^ Hemorrhoidectomy versus Conventional Hemorrhoidectomy: Comparison in Outcome. Open Access Maced. J. Med. Sci..

[4] Wei D., Jiang P., Gao R., Zhao Y. (2023). Prevention and Treatment of Anastomotic Strictures After Procedure for Prolapse and Hemorrhoids. Risk Manag. Healthc. Policy.

[5] Durgun C., Yiğit E. (2023). Laser Hemorrhoidoplasty Versus Ligasure Hemorrhoidectomy: A Comparative Analysis. Cureus.

[6] Gardner I. (2019). Benign Anorectal Disease: Hemorrhoids, Fissures, and Fistulas. Ann. Gastroenterol..

[7] Elnaim A.L.K., Musa S., Wong M.P.-K., Sagap I. (2021). A Prospective Interventional Study on LigaSureTM Haemorrhoidectomy as a Daycare Procedure. Malays. J. Med. Sci..

[8] Cheng K.-C., Song L.-C., Wu K.-L., Chen H.-H., Lee K.-C. (2022). Risk Factors of Delayed Hemorrhage after LigaSure Hemorrhoidectomy. BMC Surg..

[9] Chen C.-W., Lu T.-J., Hsiao K.-H. (2021). Surgical Outcomes of LigaSure Hemorrhoidectomy in the Elderly Population: A Retrospective Cohort Study. BMC Gastroenterol..

[10] Bibi S., Saqib K., Irfan I., Tabbasam S., Ahmad H. (2023). LigaSure Hemorrhoidectomy vs Milligan Morgan Hemorrhoidectomy: A Quasi-Experimental Study. Prof. Med. J..

[11] Jin J., Unasa H., Bahl P., Mauiliu-Wallis M., Svirskis D., Hill A. (2023). Can Targeting Sphincter Spasm Reduce Post-Haemorrhoidectomy Pain? A Systematic Review and Meta-Analysis. World J. Surg..

[12] Robinson A., McIntosh J., Peberdy H., Wishart D., Brown G., Pope H., Kumar S. (2019). The Effectiveness of Physiotherapy Interventions on Pain and Quality of Life in Adults with Persistent Post-Surgical Pain Compared to Usual Care: A Systematic Review. PLoS ONE.

[13] Amir A., Nazir A., Umair A., Khan M.A., Maqbool S., Anwar M.I., Fazal F. (2023). Comparison of Pedicle Coagulation Hemorrhoidectomy With LigaSure Versus Conventional Milligan Morgan Hemorrhoidectomy in Reducing Post-Operative Pain: A Randomized Controlled Trial. Cureus.

[14] Foong D., Zhou J., Zarrouk A., Ho V., O’Connor M.D. (2020). Understanding the Biology of Human Interstitial Cells of Cajal in Gastrointestinal Motility. Int. J. Mol. Sci..

[15] Mah S.A., Du P., Avci R., Vanderwinden J.-M., Cheng L.K. (2022). Analysis of Regional Variations of the Interstitial Cells of Cajal in the Murine Distal Stomach Informed by Confocal Imaging and Machine Learning Methods. Cell. Mol. Bioeng..

[16] Mah S.A., Avci R., Cheng L.K., Du P. (2021). Current Applications of Mathematical Models of the Interstitial Cells of Cajal in the Gastrointestinal Tract. WIREs Mech. Dis..

[17] Lu P., Lifshitz L.M., Bellve K., ZhuGe R. (2024). TMEM16A in Smooth Muscle Cells Acts as a Pacemaker Channel in the Internal Anal Sphincter. Commun. Biol..

[18] Le S.C., Yang H. (2020). An Additional Ca^2+^ Binding Site Allosterically Controls TMEM16A Activation. Cell Rep..

[19] Kather J.N., Pearson A.T., Halama N., Jäger D., Krause J., Loosen S.H., Marx A., Boor P., Tacke F., Neumann U.P. (2019). Deep Learning Can Predict Microsatellite Instability Directly from Histology in Gastrointestinal Cancer. Nat. Med..

[20] L’Imperio V., Wulczyn E., Plass M., Müller H., Tamini N., Gianotti L., Zucchini N., Reihs R., Corrado G.S., Webster D.R. (2023). Pathologist Validation of a Machine Learning–Derived Feature for Colon Cancer Risk Stratification. JAMA Netw. Open.

[21] Najdawi F., Sucipto K., Mistry P., Hennek S., Jayson C.K.B., Lin M., Fahy D., Kinsey S., Wapinski I., Beck A.H. (2023). Artificial Intelligence Enables Quantitative Assessment of Ulcerative Colitis Histology. Mod. Pathol..

[22] Codipilly D.C., Faghani S., Hagan C., Lewis J., Erickson B.J., Iyer P.G. (2024). The Evolving Role of Artificial Intelligence in Gastrointestinal Histopathology: An Update. Clin. Gastroenterol. Hepatol..

[23] Yilmaz F., Brickman A., Najdawi F., Yakirevich E., Egger R., Resnick M.B. (2024). Advancing Artificial Intelligence Integration Into the Pathology Workflow: Exploring Opportunities in Gastrointestinal Tract Biopsies. Lab. Investig..

[24] Jocher G., Qiu J. (2024). Ultralytics YOLO11. https://docs.ultralytics.com/models/yolo11/.

[25] Sheikh P., Régnier C., Goron F., Salmat G. (2020). The Prevalence, Characteristics and Treatment of Hemorrhoidal Disease: Results of an International Web-Based Survey. J. Comp. Eff. Res..

[26] Oakland K., Chadwick G., East J.E., Guy R., Humphries A., Jairath V., McPherson S., Metzner M., Morris A.J., Murphy M.F. (2019). Diagnosis and Management of Acute Lower Gastrointestinal Bleeding: Guidelines from the British Society of Gastroenterology. Gut.

[27] Brown S.R. (2017). Haemorrhoids: An Update on Management. Ther. Adv. Chronic Dis..

[28] Kibret A.A., Oumer M., Moges A.M. (2021). Prevalence and Associated Factors of Hemorrhoids among Adult Patients Visiting the Surgical Outpatient Department in the University of Gondar Comprehensive Specialized Hospital, Northwest Ethiopia. PLoS ONE.

[29] Onder T., Altiok M. (2023). A Retrospective Comparative Study of Hemorrhoidal Artery Ligation versus Ligasure Hemorrhoidectomy for the Third Degree Hemorrhoidal Disease. Asian J. Surg..

[30] Prokopakis E.P., Lachanas V.A., Vardouniotis A.S., Velegrakis G.A. (2010). The Use of the Ligasure Vessel Sealing System in Head and Neck Surgery: A Report on Six Years of Experience and a Review of the Literature. B-ENT.

[31] McGenity C., Clarke E.L., Jennings C., Matthews G., Cartlidge C., Freduah-Agyemang H., Stocken D.D., Treanor D. (2024). Artificial Intelligence in Digital Pathology: A Systematic Review and Meta-Analysis of Diagnostic Test Accuracy. Npj Digit. Med..

[32] Lohsiriwat V., Jitmungngan R. (2022). Strategies to Reduce Post-Hemorrhoidectomy Pain: A Systematic Review. Med. Kaunas Lith..

[33] Huang Y.-J., Chen C.-Y., Chen R.-J., Kang Y.-N., Wei P.-L. (2018). Topical Diltiazem Ointment in Post-Hemorrhoidectomy Pain Relief: A Meta-Analysis of Randomized Controlled Trials. Asian J. Surg..

[34] Nagaty M. (2022). A Comparison between the Outcome of LigaSure Hemorrhoidectomy Versus Conventional Milligan Morgan’s Technique. Al-Azhar Int. Med. J..

[35] Uzzaman M.M., Siddiqui M.R.S. (2011). A Brief Literature Review on the Management of Post-Haemorrhoidectomy Pain. Surg. Tech. Dev..

[36] Emile S.H. (2019). Evidence-Based Review of Methods Used to Reduce Pain after Excisional Hemorrhoidectomy. J. Coloproctology.

[37] Perera N., Liolitsa D., Iype S., Croxford A., Yassin M., Lang P., Ukaegbu O., Van Issum C. (2012). Phlebotonics for Haemorrhoids. Cochrane Database Syst. Rev..

[38] Orefice R., Litta F., Parello A., De Simone V., Campennì P., Marra A.A., Ratto C. (2021). A Prospective Study on the Efficacy of Two Different Phlebotonic Therapies as a Bridge to Surgery in Patients with Advanced Hemorrhoidal Disease. J. Clin. Med..

[39] Al-Sajee D., Huizinga J.D. (2012). Interstitial Cells of Cajal: Pathology, Injury and Repair. Sultan Qaboos Univ. Med. J..

[40] Torihashi S., Horisawa M., Watanabe Y. (1999). C-Kit Immunoreactive Interstitial Cells in the Human Gastrointestinal Tract. J. Auton. Nerv. Syst..

[41] Brunenieks I., Pekarska K., Kasyanov V., Groma V. (2017). Biomechanical and Morphological Peculiarities of the Rectum in Patients with Obstructed Defecation Syndrome. Rom. J. Morphol. Embryol..

[42] Hagger R., Gharaie S., Finlayson C., Kumar D. (1998). Distribution of the Interstitial Cells of Cajal in the Human Anorectum. J. Auton. Nerv. Syst..

[43] Kaji N., Hori M. (2023). Interstitial Cells of Cajal in Gastrointestinal Inflammatory Diseases. J. Smooth Muscle Res..

[44] Wu C.-E., Tzen C.-Y., Wang S.-Y., Yeh C.-N. (2019). Clinical Diagnosis of Gastrointestinal Stromal Tumor (GIST): From the Molecular Genetic Point of View. Cancers.

[45] Yin J., Chen J.D.Z. (2008). Roles of Interstitial Cells of Cajal in Regulating Gastrointestinal Motility: *In Vitro versus in Vivo* Studies. J. Cell. Mol. Med..

[46] Chevalier N.R., Ammouche Y., Gomis A., Teyssaire C., De Santa Barbara P., Faure S. (2020). Shifting into High Gear: How Interstitial Cells of Cajal Change the Motility Pattern of the Developing Intestine. Am. J. Physiol.-Gastrointest. Liver Physiol..

[47] Hwang S.J., Blair P.J.A., Britton F.C., O’Driscoll K.E., Hennig G., Bayguinov Y.R., Rock J.R., Harfe B.D., Sanders K.M., Ward S.M. (2009). Expression of Anoctamin 1/TMEM16A by Interstitial Cells of Cajal Is Fundamental for Slow Wave Activity in Gastrointestinal Muscles. J. Physiol..

[48] Lees-Green R., Gibbons S.J., Farrugia G., Sneyd J., Cheng L.K. (2014). Computational Modeling of Anoctamin 1 Calcium-Activated Chloride Channels as Pacemaker Channels in Interstitial Cells of Cajal. Am. J. Physiol.-Gastrointest. Liver Physiol..

[49] Sanders K.M., Hashitani H., Lang R.J. (2019). Spontaneous Electrical Activity and Rhythmicity in Gastrointestinal Smooth Muscles. Smooth Muscle Spontaneous Activity.

[50] Forrest A.S., Hennig G.W., Jokela-Willis S., Park C.D., Sanders K.M. (2009). Prostaglandin Regulation of Gastric Slow Waves and Peristalsis. Am. J. Physiol.-Gastrointest. Liver Physiol..

[51] Huizinga J.D., Hussain A., Chen J.-H. (2021). Interstitial Cells of Cajal and Human Colon Motility in Health and Disease. Am. J. Physiol.-Gastrointest. Liver Physiol..

[52] Choi E.L., Taheri N., Tan E., Matsumoto K., Hayashi Y. (2023). The Crucial Role of the Interstitial Cells of Cajal in Neurointestinal Diseases. Biomolecules.

[53] Sweet T., Abraham C.M., Rich A. (2024). Origin and Development of Interstitial Cells of Cajal. Int. J. Dev. Biol..

[54] Strege P.R., Ou Y., Sha L., Rich A., Gibbons S.J., Szurszewski J.H., Sarr M.G., Farrugia G. (2003). Sodium Current in Human Intestinal Interstitial Cells of Cajal. Am. J. Physiol.-Gastrointest. Liver Physiol..

[55] Won K.-J., Sanders K.M., Ward S.M. (2005). Interstitial Cells of Cajal Mediate Mechanosensitive Responses in the Stomach. Proc. Natl. Acad. Sci. USA.

[56] Truong Thuy Nguyen V., Taheri N., Choi E.L., Kellogg T.A., Linden D.R., Hayashi Y. (2023). Insulin-Like Growth Factor1 Preserves Gastric Pacemaker Cells and Motor Function in Aging via ERK1/2 Activation. Cell. Mol. Gastroenterol. Hepatol..

[57] Du P., Hameed A., Angeli T.R., Lahr C., Abell T.L., Cheng L.K., O’Grady G. (2015). The Impact of Surgical Excisions on Human Gastric Slow Wave Conduction, Defined by High-resolution Electrical Mapping and in Silico Modeling. Neurogastroenterol. Motil..

[58] Camilleri M., Tack J. (2023). Is the Quantification of Interstitial Cells of Cajal in Gastric Biopsy Samples in Patients with Gastroparesis Ready for Prime Time?. Gastroenterology.

[59] Patel V., Khan M.N., Shrivastava A., Sadiq K., Ali S.A., Moore S.R., Brown D.E., Syed S. (2020). Artificial Intelligence Applied to Gastrointestinal Diagnostics: A Review. J. Pediatr. Gastroenterol. Nutr..

[60] Lecuelle J., Truntzer C., Basile D., Laghi L., Greco L., Ilie A., Rageot D., Emile J.-F., Bibeau F., Taïeb J. (2024). Machine Learning Evaluation of Immune Infiltrate through Digital Tumour Score Allows Prediction of Survival Outcome in a Pooled Analysis of Three International Stage III Colon Cancer Cohorts. eBioMedicine.

[61] Zhou J., Hu N., Huang Z.-Y., Song B., Wu C.-C., Zeng F.-X., Wu M. (2021). Application of Artificial Intelligence in Gastrointestinal Disease: A Narrative Review. Ann. Transl. Med..

