# Supplementary figures and images for: Utilisation of Deep Neural Networks for Estimation of Cajal Cells in the Anal Canal Wall of Patients with Advanced Haemorrhoidal Disease Treated by LigaSure Surgery
Source: Cells. 2025 Apr 5;14(7):550. doi: 10.3390/cells14070550 (PMC11989036; doi:10.3390/cells14070550)

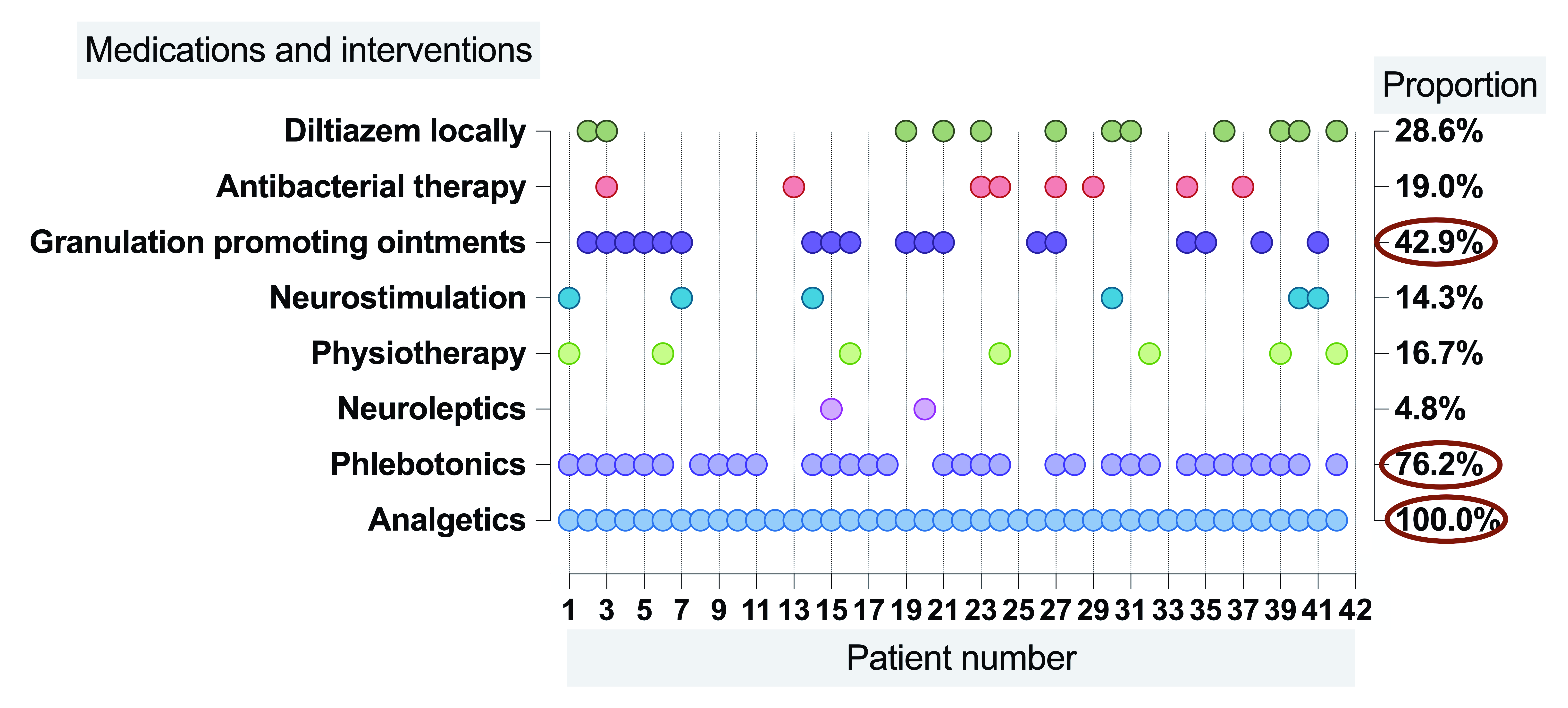

Supplement: Supplementary file 1 [file cells-14-00550-s001.zip › Supplementary Figure S1 600dpi.tiff]

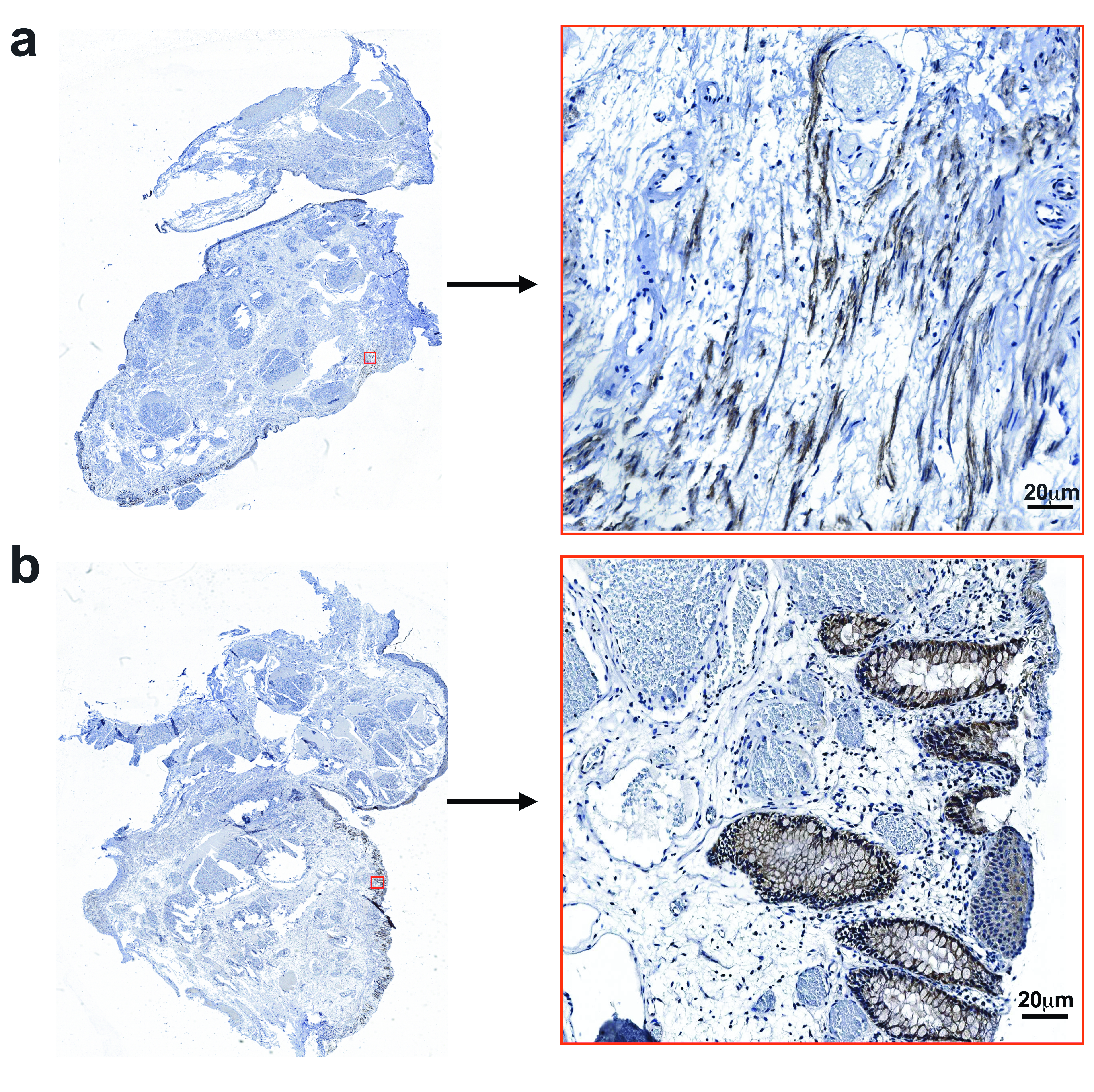

Supplement: Supplementary file 1 [file cells-14-00550-s001.zip › Supplementary Figure S2 600dpi.tiff]

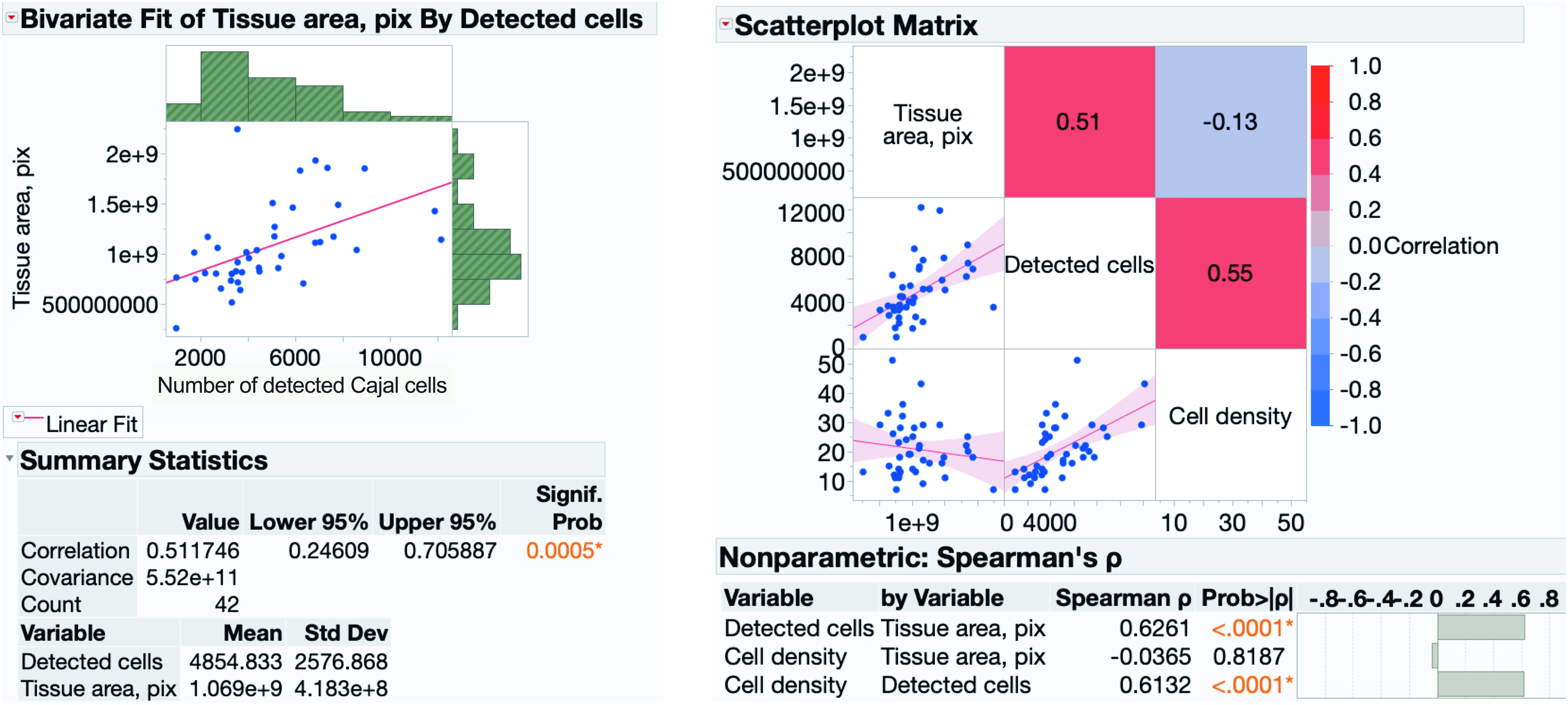

Supplement: Supplementary file 1 [file cells-14-00550-s001.zip › Supplementary Figure S4 600dpi.tiff]
